# Advancement in Solubilization Approaches: A Step towards Bioavailability Enhancement of Poorly Soluble Drugs

**DOI:** 10.3390/life13051099

**Published:** 2023-04-27

**Authors:** Lakshmi Kumari, Yash Choudhari, Preeti Patel, Ghanshyam Das Gupta, Dilpreet Singh, Jessica M. Rosenholm, Kuldeep Kumar Bansal, Balak Das Kurmi

**Affiliations:** 1 Department of Pharmaceutics, ISF College of Pharmacy, GT Road, Moga 142001, Punjab, India; lakshmikumari2473@gmail.com (L.K.); yashjat054@gmail.com (Y.C.); drgdg@rediffmail.com (G.D.G.); dilpreet.daman@gmail.com (D.S.); 2 Department of Pharmaceutical Chemistry, ISF College of Pharmacy, GT Road, Moga 142001, Punjab, India; 3 Pharmaceutical Sciences Laboratory, Faculty of Science and Engineering, Åbo Akademi University, 20520 Turku, Finland; jessica.rosenholm@abo.fi

**Keywords:** solubility, bioavailability, prodrug, oral drug delivery, nanotechnology, solid dispersion, supercritical fluid technology, liposomes, dendrimer, micelles, polymeric micelles, MOFs, carbon nanotubes, nanogels, nanoemulsions, mesoporous silica nanoparticles

## Abstract

A drug’s aqueous solubility is defined as the ability to dissolve in a particular solvent, and it is currently a major hurdle in bringing new drug molecules to the market. According to some estimates, up to 40% of commercialized products and 70–90% of drug candidates in the development stage are poorly soluble, which results in low bioavailability, diminished therapeutic effects, and dosage escalation. Because of this, solubility must be taken into consideration when developing and fabricating pharmaceutical products. To date, a number of approaches have been investigated to address the problem of poor solubility. This review article attempts to summarize several conventional methods utilized to increase the solubility of poorly soluble drugs. These methods include the principles of physical and chemical approaches such as particle size reduction, solid dispersion, supercritical fluid technology, cryogenic technology, inclusion complex formation techniques, and floating granules. It includes structural modification (i.e., prodrug, salt formation, co-crystallization, use of co-solvents, hydrotrophy, polymorphs, amorphous solid dispersions, and pH variation). Various nanotechnological approaches such as liposomes, nanoparticles, dendrimers, micelles, metal organic frameworks, nanogels, nanoemulsions, nanosuspension, carbon nanotubes, and so forth have also been widely investigated for solubility enhancement. All these approaches have brought forward the enhancement of the bioavailability of orally administered drugs by improving the solubility of poorly water-soluble drugs. However, the solubility issues have not been completely resolved, owing to several challenges associated with current approaches, such as reproducibility in large scale production. Considering that there is no universal approach for solving solubility issues, more research is needed to simplify the existing technologies, which could increase the number of commercially available products employing these techniques.

## 1. Introduction

Solubility is the phenomenon of dissolving a solute in a solvent, which is essential to produce a homogenous system. In quantitative terms, solubility may be defined as the required strength of the solute dissolved in a solution at a given pH, temperature, and pressure [1]. In contrast, in qualitative terms, solubility is the material’s ability to be melted in a saturated solution at a specific temperature [2,3]. Solubility is presented with numerous terminologies such as molality, volume fraction, parts of solvent, percentage, molarity, mole fraction, and so forth [4]. US Pharmacopoeias define solubility as the milliliters of solvent necessary to dissolve one gram of solute [5]. Solubility is standardly determined using two approaches: thermodynamic solubility and kinetic solubility. The main distinction between the two methods is that the solid compound is added to the aqueous medium to determine thermodynamic solubility, whereas the pre-dissolved compound is used as the initial substance to determine kinetic solubility. Thermodynamic solubility provides a response to the question: “How much does the substance dissolve?” Conversely, kinetic solubility answers: “How much does the molecule precipitate?” [6]. It is obvious that thermodynamic solubility plays a vital role in the solubility determination of poorly soluble drugs. In addition, dissolution is dependent on thermodynamic solubility. Notably, one should distinguish between the terms ‘dissolution’ and ‘solubility’. When a solute in any phase, either the gaseous, liquid, or solid phase, dissolves in a solvent to create a solution, the term “dissolution” is used. In contrast, the term “solubility” refers to the highest concentration of a solute that may dissolve in a solvent at a specific temperature [7].

**Figure 1:**
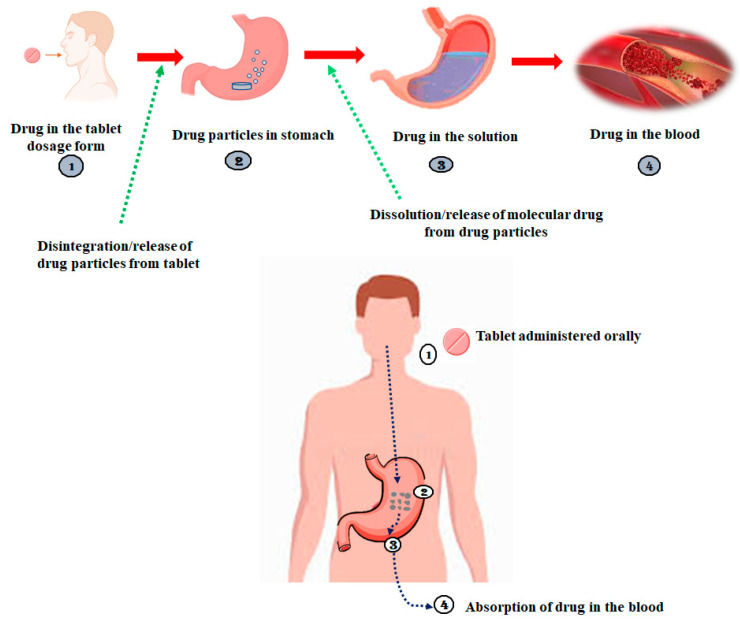
Fate of drug molecule after administering via oral route.

Drugs administered via the oral route in a solid dosage form are first disintegrated into smaller parts or even primary particles, from which the drug molecules are freer to dissolve in the gastrointestinal tract (GIT) fluids than from an intact tablet; the molecular dissolution of the drug is then followed by its penetration through the intestinal barrier, as displayed in Figure 1 [8]. Given that all bodily fluids are water-based solutions, aqueous solubility is an essential criterion to achieve the appropriate concentrations of the drug molecules in the systemic circulation to elicit the required therapeutic efficacy. If a drug molecule has very low solubility, it cannot be dissolved in the GIT fluids, which hinders its permeability and, thus, bioavailability because it is directly related to the drug solubility. Low bioavailability observed with poorly soluble drugs make the final formulation expensive because high doses are needed to obtain therapeutic benefits and, sometimes, they might cause toxicity [9,10].

Depending on the solubility and permeability in the GIT, drug substances are categorized in four BCS classes (biopharmaceutical classification system, as listed in Table 1) [10,11]. Because of low solubility, despite high permeability, BCS class II drugs are associated with a slower dissolution rate in the GI tract, leading to low bioavailability. Owing to low aqueous solubility, a small concentration gradient between the intestine and the bloodstream results in restricted transport across biological membranes and, consequently, poor absorption is often reported. In contrast, in addition to low aqueous solubility, BCS-class IV drugs also have low permeability, which reduces their ability to be absorbed. However, BCS class IV drugs sometimes make poor drug development candidates due to limited membrane permeability since solubility and dissolution augmentation may not be sufficient to increase their bioavailability. However, these types of compounds cannot be neglected only because of their permeability difficulties. Therefore, class IV compounds may be developed via the current methods utilized for BCS class II drugs along with absorption enhancers. In the lead optimization stage, choosing a better drug candidate with more suitable physiochemical characteristics is another formulation development strategy for class IV drugs [12]. The development of various techniques to address unsatisfactory biopharmaceutical properties and the advancement of knowledge in the field of drug delivery systems for oral administration were both influenced by the need for efficient formulations for BCS-classes II and IV drugs. It has even been estimated that up to 90% of new molecular entities fall in BCS classes II and IV. It has been reported that only eight percent of novel drug candidates currently exhibit excellent permeability and solubility. Water-insoluble or poorly water-soluble medications account for more than 1/3 of the pharmaceuticals classified in the US Pharmacopeia [13]. Recently, it was claimed that around half of all drug molecules failed during the development stage due to poor aqueous solubility. Lead compounds with poor solubility characteristics resulted in inefficient absorption from the administration site, resulting in a higher rate of therapeutic loss due to poor pharmacokinetics [14].

The primary goal of the formulating R&D divisions of a pharmaceutical company is to make the medication accessible at the correct place within the body for its foreseen activity and in the most effective dosage [14]. The most challenging attribute in the field of drug discovery is improving drug solubility. For the goal of improving the solubility of poorly water-soluble drugs, a number of strategies have been described in the literature. These methods are preferred based on particular characteristics, such as the properties of the drug under consideration, intended dosage form types, and excipient properties [15,16]. In light of this, we aim to focus on the numerous techniques utilized to increase the solubility and, ultimately, bioavailability of poorly water-soluble drugs in this review. The various conventional methods for solubility enhancement, such as inclusion complex formation techniques, supercritical fluid technology (SCF), cryogenic technology, particle size reduction, and solid dispersion, are discussed. The conventional approaches include micronization, the use of penetration enhancers or co-solvents, the surfactant dispersion method, salt formation, precipitation, and other methods. However, the effectiveness of these methods in improving the solubility of poorly soluble drugs is still limited. Other methods include vesicular systems such as solid dispersion and cyclodextrin inclusion complexes, which have shown promise as drug delivery systems but have the significant drawbacks including the inability to be applied on all drugs [17,18]. Simultaneously, different nanotechnological approaches for solubility enhancement are also discussed in detail, which include dendrimers [19,20,21], micelles [22,23,24,25], solid lipid nanoparticles [26,27], liposomes [28,29], and polymeric nanoparticles [30]. For almost 30 years, nanotechnological products have been in clinical use within the healthcare industry.

**Figure 2:**
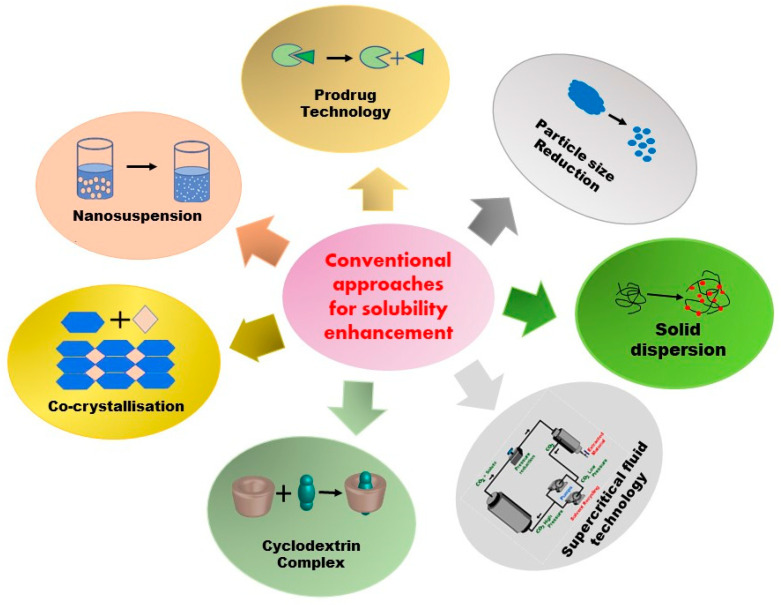
Conventional methods for solubility enhancement.

**Figure 3:**
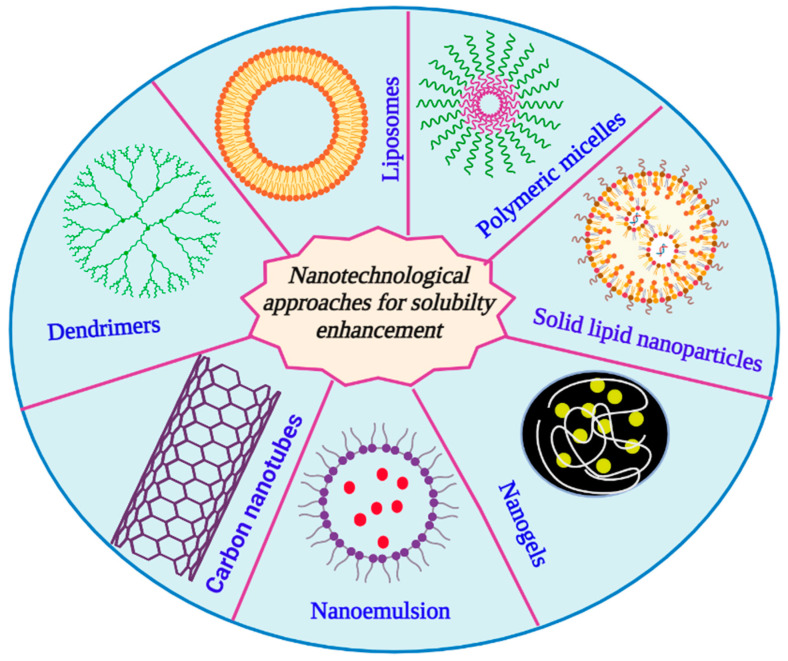
Nanocarrier-mediated solubility enhancement techniques.

In 2000, the US FDA authorized the first medication that increases solubility using nanotechnology. Elan created a nanoparticulate version of Wyeth’s Rapamune (sirolimus Wyeth-Ayerst, Philadelphia, PA, USA), an immunosuppressive medication used to prevent organ transplant rejection, and it has since become the fastest-selling medication in the transplant market [31]. Similarly, numerous clinical products, such as Doxil^®^, Ambisome^®^, and DepoDur^TM^, are examples of how liposomes have had a substantial impact on drug delivery systems in the healthcare industry [32]. During the COVID-19 outbreak, the advantage of nanotechnological approaches became more widely evident for the public. The first two COVID-19 messenger RNA (mRNA) vaccines by Pfizer/BioNTech and Moderna, approved in December 2020, were developed with unmatched speed and perhaps received the most positive media coverage out of all the COVID-19 vaccines to date. Undoubtedly, they have demonstrated notable effectiveness in disease prevention and represent the most recent successful application of lipidic nanoparticles as a delivery system [33]. Despite not being examples of solubility enhancement, similar approaches can be utilized for these purposes as well. The various types of solubility enhancement strategies are summarized in Figure 2 and Figure 3, and Table 2 presents the factors affecting solubilization.

### Review Highlights

- The aqueous solubility of a drug plays a crucial role in drug dissolution and release, absorption, and, consequently, bioavailability.
- Conventional approaches, which include particle size reduction, solid dispersion, co-crystallization, prodrug approach, supercritical fluid technology, and inclusion complex, have been in use for decades for the enhancement of the aqueous solubility of poorly soluble drugs.
- Nanotechnology has the potential to revive poorly performing marketed drugs and many of those pre-clinically promising candidates that were “shelved” due to inadequate water-solubility.
- A variety of nanocarriers have been utilized and are still at the development stage. These include the dendrimers, micelles, SLNs, MOFs, CNTs, nanogels, and mesoporous silica nanoparticles used to increase the bioavailability of poorly soluble drugs; they could be useful for the future formulation of development research.

## 2. Conventional Approaches

Conventional approaches have been in use for decades for the enhancement of the aqueous solubility of poorly soluble drugs. Micronization, solid dispersion, prodrug, cyclodextrin inclusion complexes, supercritical fluid technology, and cryogenic technology are strategies that fall under the category of conventional approaches (Figure 2).

### 2.1. Particle Size Reduction

The primary molecular size of the drug powder has a direct impact on the bioavailability of poorly soluble drugs. The reduction in particle size leads to an increase in surface area, which further improves the dissolution properties due to the increased contact area with the solvent. In addition, particle size reduction allows the rapid diffusion of the solvent. Milling techniques, such as, jet mills, rotor-stator colloid mills, and other types of mills, reduce the particle size of drug raw materials [12,40]. However, thermal stress should be considered when using spray drying for thermosensitive substances [41]. Micronization techniques can convert particles into sizes of less than 5 μm in diameter and yield uniform particle sizes. Various types of micronization techniques, such as milling, supercritical fluid technology, microprecipitation and microcrystallization, and spray freezing into liquid, affect the characteristics of the micronized drug substance [42].

### 2.2. Cyclodextrin Inclusion Complexes

Inclusion complexes are formed by inserting a non-polar molecule (guest molecule) into the cavity of another molecule or group of molecules (host molecule). The inclusion complex creation approach has been used more accurately than any other solubility enhancement method to increase the aqueous solubility, dissolution rate, and bioavailability of the drugs. Here, cyclodextrins (CDs) have been used as the most common host molecule. Poorly soluble therapeutics can have their physicochemical and biological characteristics changed with CDs by having drug molecules included in the cavity of the disc. CDs can attach lipophilic compounds via a variety of intermolecular interactions because of the hollow, lipophilic core cavity [43]. The kneading method, physical mixing, the co-precipitation method, and the solvent evaporation method are widely used for the preparation of inclusion complexes [44]. Rivaroxaban (RIV), an oral anticoagulant, is a poorly soluble drug having a solubility of 0.005 and 0.006 mg/mL in water and acetate buffer of pH 4.5, respectively. Sherje et al. formulated rivaroxaban-loaded β-Cyclodextrin-based inclusion complexes. They have developed RIV inclusion complexes via the kneading method, spray drying, and physical mixing, which showed an increased solubility in water by 3.36-, 2.34-, and 4.02-fold; increments of 1.88-, 3.68-, and 1.78-fold were obtained in the acetate buffer [43].

### 2.3. Solid Dispersions

For oral dosage forms, solid dispersions (SD) have been a good technique for enhancing drug solubility, absorption, and therapeutic efficacy [45]. SD is a group of solid materials with at least two distinct components: a hydrophilic matrix and a hydrophobic drug. The molecular dispersion of one or more hydrophobic drugs in a hydrophilic carrier matrix is referred to as solid dispersion [46,47]. Formulating solid dispersions is a method of choice within pharmaceutical industries for improving drug solubility in the dosage form. Some hydrophilic carriers used to create solid dispersions are polyvinylpyrrolidone (povidone, PVP), polyethylene glycols (PEGs), hydroxy propyl methyl cellulose (HPMC), and Plasdone-S630. Surfactants such as sodium lauryl sulphate (SLS), docusate sodium, Pluronic-F68, Myrj-52, and Tween-80 are used in solid dispersion formulation. Sekiguchi and Obi explored the manufacturing or dissolution properties of eutectic melts containing a sulphonamide drug and a water-soluble carrier in the mid-1960s [48]. Solid dispersion using appropriate hydrophilic carriers has been shown to improve the solubility of celecoxib, halofantrine, and ritonavir. Hydrophobic drugs could be formulated into solid dispersions using many methods to increase their water solubility [49]. There are many commercialized products available in the market that are based on solid dispersions for delivering poorly soluble drugs; some examples are listed in Table 3.

In a study by Muniandy et al., hyper-branched poly (glycerol ester amide) (HPGEA) with an average molecular weight of 5000–12,000 Da and a degree of branching of approx. 60% was used as a drug carrier in the fusion–solvent process to formulate lovastatin solid dispersions (LOV SD). SD having LOV:HPGEA = 5:95% w/w demonstrated significant improvement in in vitro dissolution, and the same formulation achieved a more than 2-fold increase in cumulative drug release and a more than 3-fold increase in solubility over pure LOV [50]. Various methods for preparing solid dispersions are discussed below:

Hot melt method (fusion method): A drug and a water-soluble carrier are heated until they melt. Under vigorous stirring, the liquefied fluid is quickly cooled and hardened on an ice bath. The resulting solid bulk is crushed, pulverized, and sieved, and tableting agents are then used to compress these into tablets [51]. One major limitation is that many drug molecules may degrade under elevated temperatures [41].

Hot-melt extrusion: Hot-melt extrusion is similar to fusion except that the extruder causes a strong mixing of the components. The miscibility of medicines and the matrix can be an issue in the classic fusion method, leading to the non-uniform distribution of the drug. High shear forces in the extruder cause a rise in local temperature, which is an issue for heat-sensitive materials. Nevertheless, opposed to the old fusion approach, this method enables continuous production, making it suitable for large-scale manufacturing [15]. To improve the dissolution and oral bioavailability of oleanolic acid, a solid dispersion of oleanolic acid with a carrier consisting of PVP VA 64 polymer was developed, and it was found that the drug dissolution of prepared SD was better (i.e., approx. 90% of drug released in 10 min) compared to the free drug (i.e., 37% in 2 h) and the physical mixture (i.e., 45% in 2 h) [52].

Solvent evaporation method: The solvent evaporation method is the foremost applicable method for improving the solubility of poorly water-soluble drugs, especially for thermolabile components. Unlike the melting method, where heat is used for the mixing of the drug and carrier, this method allows the mixing of the drug and carrier with the aid of a solvent, which also provides an advantage for using carriers with extremely high melting points. In order to achieve homogeneous mixing, the drug, as well as the carrier, are both dissolved in a volatile solvent, followed by the formation of solid dispersions after evaporating the solvent via constant stirring. The solubility of various poorly soluble anti-cancer drugs, such as paclitaxel, docetaxel, everolimus, and exemestane, has been increased by this method [53]. Chen and his colleagues found in their studies that both the solubility and dissolution of docetaxel had improved in solid dispersion formulation. They found that, in comparison to the free drug, the dissolution and solubility of docetaxel-loaded emulsified solid dispersion improved 12.7-fold and 34.2-fold, respectively at 2 h [54].

Spray drying: The spray drying technique for the development of solid dispersions involves the preparation of a feed solution in which the carrier has to be dissolved in water, while the drug molecule has to be dissolved in the required solvent, followed by sonication. Afterwards, fine droplets of drug molecules are formed through a nozzle using high pressure in a drying chamber. Herbrink et al. developed a solid dispersion of nilotinib with the aid of the spray drying technique to improve solubility, and the results showed that, compared to free nilotinib, the solubility of spray dried nilotinib SD had improved by 630 times at a drug: Soluplus^®^ (BASF SE-Ludwigshafen, Germany) ratio of 1:7 [55].

Owing to several benefits, solid dispersions were frequently used to improve the drug’s water solubility. Drugs interacting with hydrophilic carriers can reduce agglomeration and release in a supersaturation state, resulting in quick absorption and improved bioavailability. This is one of the most significant benefits of solid dispersion. Further, when compared to other forms, such as liquid products, solid dispersion can be created as a solid oral dosage form, which is more feasible for patients [53]. Despite having a number of advantages, solid dispersions exhibit physical instability, vary in crystallinity with time, and are sensitive to temperature and humidity on storage due to their thermodynamic instability. These substances can hasten the phase separation and crystallization of solid dispersions by increasing molecular mobility generally, lowering the glass transition temperature (Tg), or interfering with interactions between the drug and carrier, which decreases the drug’s solubility and rate of dissolution. The effectiveness of the therapy and the quality of the medications may be affected by the stability of solid dispersions during storage [53,56,57].

### 2.4. Prodrugs

**Figure 4:**
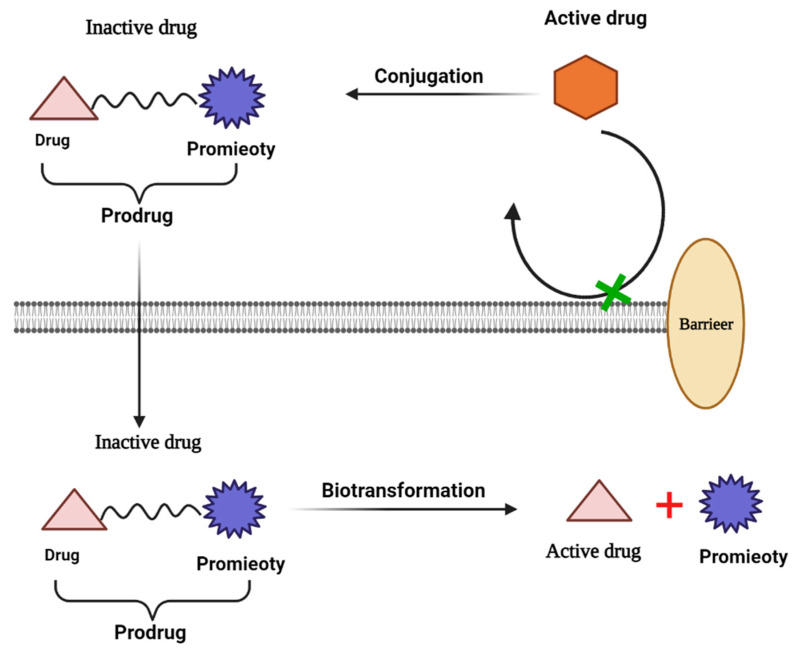
Mechanism of prodrug solubility enhancement.

A prodrug is an inactive, chemically modified parent drug that has enhanced aqueous solubility and can be converted into the active parent drug via rapid biotransformation. The use of prodrugs can also enhance pharmaceutical qualities such as odor, taste, and chemical stability and alleviate the irritation and pain associated with pharmaceuticals and problems in the preparation or manufacture of the API. In addition, such prodrugs can lead to pharmacokinetic profile optimization and decrease or remove the first-pass effect [58]. The two major prodrug formulation groups to consider are: (i) carrier-linked prodrugs, in which the parent drug is chemically connected to a prodrug molecule, and (ii) bio precursor prodrugs. Carrier-linked prodrugs were classified as bipartite prodrugs because the carrier is attached to the parent drug directly, or tripartite prodrugs, wherein the carrier is linked to the parent drug by a spacer, as presented in Figure 4 [59,60].

Classic prodrugs are described as carrier-linked prodrugs. Mixed prodrugs, as in traditional prodrugs, are latent forms in which the carrier has bioprecursor properties and is linked to a drug. The drug is released after the bond has been cleaved via an enzymatic reaction. This prodrug form is also known as a CDS (chemical delivery system). Mutual prodrugs, such as classic prodrugs, contain a pharmacologically active carrier, enabling the development of a prodrug either with different or similar therapeutic activities, operating via distinct and similar mechanisms of action [60].

Some prodrugs do not have an apparent carrier or promoter but instead reemerge out of a molecular change in the actual prodrug, resulting in a novel active molecule. According to US-FDA, among all new drug molecules approved, 12.4% were prodrugs between 2008 and 2017 [61]. To tackle the poor oral bioavailability of a poorly soluble but highly permeable HCV NS5B polymerase inhibitor, a prodrug methodology was introduced. Microsomes from the liver or the intestinal tissues, plasma, simulated gastric fluids, and simulated intestinal fluids were used in a series of in vitro assays to assess the bioconversion rates of structurally diverse prodrug derivatives. The prospective candidates’ in vivo bioconversion was evaluated after orally administering them to rats. The original medication’s carboxylic acid component might have been converted to glycolic amide esters, which would have boosted solubility in the lipid-based self-emulsifying drug delivery system (SEDDS). When compared to parent and cross-species bioconversions, the crystalline prodrug counterpart displayed preferable solubility in certain SEDDS components, which matched the in vitro stability in liver microsomes [62].

### 2.5. Co-Crystallization

A co-crystal is a crystalline structure wherein specific stoichiometric amounts of noncovalent forces hold two or more electrically neutral substances together [63]. The co-crystallization of two active drug products, aspirin and acetaminophen, was already recorded [64]. It is similar to salt production, especially in the case of neutral substances, and can be produced via evaporation, sublimation, melt growth, and slurry preparation [40]. In one study, the formation and characterization of three different ezetimibe crystals (utilizing methylparaben as a conformator via three different processes: solution crystallization, liquid-assisted crushing, and reaction crystallization) has been reported. Differential scanning calorimetry (DSC), Fourier transform infrared (FTIR), Raman spectroscopy, and powder X-ray diffraction (PXRD) studies show that different peak melting temperatures were observed in the three co-crystals, suggesting the development of a new solid phase. The recent substantial form is caused by a low electrostatic interaction mediating the medicine and the co-former. The crystal habituation of both the drug and the co-former has been changed, respectively. The equilibrium solubility and dissolution studies of the co-crystals show that the co-crystals of ezetimibe and methylparaben could be a possible and potential alternative and effective strategy for increasing solubility [65].

### 2.6. Supercritical Fluid Technology (SCF)

Being non-toxic, non-reactive, non-flammable, and non-polluting, the implementation of SCF technology has garnered the attention of many researchers. This green technology approach has the potential to make a significant difference in the pharmaceutical industry by overcoming the limitations of several conventional processes, such as spray drying and others [66,67]. The US-FDA recognizes CO_2_ as a safe supercritical solvent and the most used supercritical solvent in the pharmaceutical manufacturing industry [68,69]. Most of the solvents employed in the preparation of the soluble versions of drugs are toxic; supercritical fluid technology can be employed to avoid this disadvantage [70]. The supercritical solution process and the residue with the compression antisolvent precipitation (PCA) are two techniques that may be used. Based on particle generation conditions (i.e., solute and solvent), there are various methods for enhancing the solubility by SCF. For solvent molecules, the rapid expansion of a supercritical solution (RESS), the rapid expansion of a supercritical solution into a liquid solvent (RESOLV), the rapid expansion of a supercritical solution into an aqueous solution (RESS-AS), and the rapid expansion of a supercritical solution with a non-solvent (RESS-N) are the various processes; gas-saturated solutions (PGSS) and the depressurization of an expanded liquid organic solution (DELOS) are the processes for solute molecules under SCF for improving solubility [71]. Jia et al. developed aescin nanoparticles with SCF using a solution-enhanced dispersion method, and they found that, compared to raw aescin, the dissolution of aescin nanoparticles was amplified approx. 5.5-fold [72]. Similar to this, resveratrol’s solubility was increased by around 2.8 times and its dissolution rate by about 1.8 times using solution-enhanced dispersion via supercritical fluids micronization [73]

A brief assessment of the advantages and disadvantages of various conventional and nanotechnological solubility enhancement techniques has been elaborated in Table 4.

## 3. Nanotechnological Approaches for Solubility Enhancement

Nanotechnology has the potential to revitalize both poorly performing marketed drugs and many of those pre-clinically promising candidates that were “beached” due to inadequate water solubility, in addition to novel therapeutic developments using components in the 1–100 nanometer range [31]. As a result of recent developments in nanotechnology, researchers have been tackling this problem by formulating drugs with the aid of nanocarriers. The most commonly utilized nanotechnology-based approaches for developing drug delivery systems include nanoemulsions, dendrimers, micelles, liposomes, solid lipid nanoparticles, polymeric nanoparticles, inorganic nanoparticles, carbon nanotubes, MOFs, and so forth [82]. Numerous studies have demonstrated that, via a variety of methods, nanoparticles can increase the oral bioavailability of hydrophobic and hydrophilic drugs [83]. In addition, there are several oral nanosuspension-based products that enhance drug absorption and dissolution available on the market [84]. A poorly water-soluble substance is made more soluble by the addition of surface-active agents in the process of solubilization. The process of solubilization involves adding an extra amphiphilic component to a material that is typically insoluble or just sparingly soluble in a particular solvent to create a thermodynamically stable solution. The dissolution of poorly soluble drugs may be achieved by the use of micellar solubilization. The inclusion of drug molecules that are poorly water-soluble is made possible by the micelles’ fluctuating polarity, which leads to solubilization, or an increase in the drug’s apparent aqueous solubility [85,86].

Nanocarriers have been widely investigated for oral drug delivery because they can prevent the drug from being enzymatically and hydrolytically degraded in the GI tract, increase the duration the drug spends in the gut through mucoadhesion, and significantly increase the drug’s absorption and bioavailability [9]. As previously stated in the introduction, drug absorption occurs after oral administration when the drug is dissolved from the formulation into aqueous gastrointestinal fluid and subsequently transported over the GI epithelium into the bloodstream. An increase in the area under the blood concentration–time curve (AUC), an increase in the maximum plasma concentration (C_max_), and a decrease in the time to maximum plasma concentration are just a few examples of how the formulation of drug nanocarriers can significantly improve the bioavailability of orally administered poorly soluble drugs (T_max_) [87,88]. Once these drug-entrapped NPs were administered orally, an increase in bioavailability has been observed. When it comes to anti-cancer drugs, for instance, it has been reported that the extremely insoluble drug paclitaxel’s in vivo bioavailability was enhanced 10-fold, when compared to Taxol taken orally, when delivered in the form of a nanoparticle. This observation indicated that higher water solubility may have an impact on the effect of enhancing the oral bioavailability of paclitaxel [89]. Several nanocarriers used for the solubility enhancement of poorly soluble drugs are briefly described below and in Table 5.

### 3.1. Liposomes

**Figure 5:**
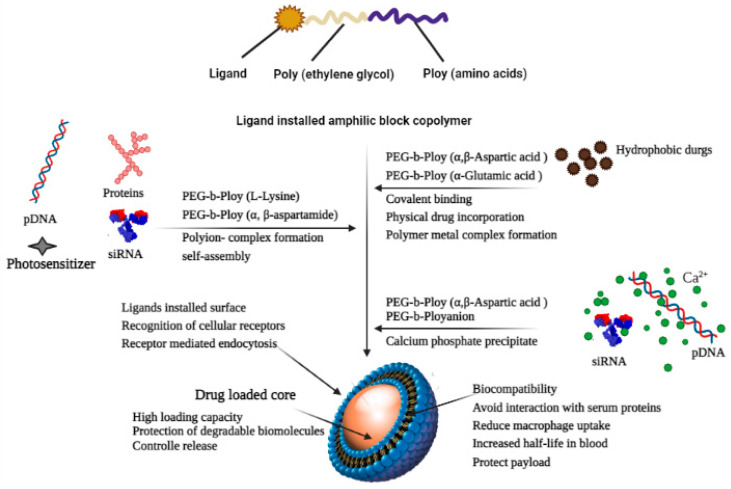
Mechanism of drug’s solubilization with the aid of liposomes.

Liposomes have been demonstrated to be one of the most promising drug delivery methods. The first liposomal (intravenous) formulation came out on the market in 1995 (Doxil^®^) and has been researched since then and integrated with various active molecules, including peptides and proteins [90]. Liposomes are created using amphipathic compounds, typically lipids; the synthesis process can be tweaked to control their size and form [91]. Liposomes are vesicles surrounded by phospholipid bilayers that can solubilize drugs that are insoluble in water in the lipid domain of the liposomal membrane [92]. The structural and compositional similarities of liposomes to biological membranes have encouraged their usage for the non-invasive oral delivery of weakly permeable drugs aided by their dissolving capability and biocompatibility [93,94]. Because liposomes may solubilize weakly water-soluble pharmaceuticals, safeguard the drug from GI tract degradation, and improve permeability across the epithelial cell membrane (boosting oral bioavailability), liposomal administration appears promising for the oral delivery of hydrophobic drugs [94]. For oral administration, liposomal membrane formulation can delay or regulate the drug’s release from the liposomes, resulting in a range of absorption rates. Bypassing the hepatic first-pass effect, liposomes can directly deliver the drug through the lymphatic route. By minimizing direct interaction with the intestinal environment by encapsulating into liposomes, drug-induced GI irritation may also be decreased [94,95]. They were first employed in the 1960s to examine biological membranes. Since then, their use has expanded to drug administration, cosmetic formulations, or the food industry, among other applications, as shown in Figure 5.

Rao et al. has prepared a liposomal drug delivery system for the enhancement of the solubility and bioavailability of Efavirenz. Efavirenz, having poor aqueous solubility (0.0085 mg/mL) and high lipophilicity (log P: 5.4), belongs to BCS class II. It has been found in their studies that there is an improvement in the solubility of Efavirenz with increasing concentration of soya lecithin in the liposomal formulation (i.e., after the addition of 900 mg of soya lecithin) along with the drug and water; solubility went up to 27.82 ± 2.55 µg/mL. In addition, according to the in vivo pharmacokinetic study, it has been reported that the oral bioavailability of the liposomal formulation has increased 2-fold compared to the free drug [96]. To overcome the poor aqueous solubility of Apigenin, Telang et al. developed the phospholipid complex of apigenin (APLC) via the incubation of phospholipon 90H along with apigenin in a solution of 1,4-dioxane and methanol at 50 °C for 2 h and then redissolved in chloroform and methanol. This mixture was further precipitated in hexane followed by vacuum drying. It was found in their study that the newly formed complex showed an increase in solubility that may be because of the amorphous state of apigenin in the APLC complex. Namely, APLC showed a 37-fold solubility improvement in the water of apigenin (i.e., from 0.62 ± 0.88 µg/mL to 22.80 ± 1.40 µg/mL) [97].

### 3.2. Dendrimers

Dendrimers, a new class of polymers, possess excellent potential for drug solubility enhancement [98,99]. Dendrimers are made up of four domains: a central core, internal layers made up of repeating units that link to the vacant spaces, external surface groups, and core (generation, G) [100]. Dendrimers containing poly (amidoamine) (PAMAM) are the most extensively studied dendrimers as drug delivery systems. They consist of an ethylenediamine core and branched units made of methyl acrylate and ethylenediamine [101]. The capacity of these hyper-branched, mono-dispersed molecules to covalently bind drug molecules to their peripheral branches and encapsulate them within the dendritic structure is unique. Several published research studies have successfully employed dendrimers to increase the solubility of poorly soluble drugs. Using physical encapsulation or covalent conjugation, dendrimers may also increase the solubility of hydrophobic compounds [21,98]. As per literature, G0 PAMAM dendrimers can greatly improve the solubility of aceclofenac, a practically water insoluble anti-inflammatory drug [102]. According to the research by Patel et al., the solubility improvement was concentration-dependent and depended on the pH, concentration, temperature, and dendrimer generation. The solubility was improved in the following sequence via dendrimer synthesis at a constant pH: G3 > G2 > G1 > G0. The enhancement in aceclofenac solubility caused by the dendrimer pH may be the result of an electrostatic interaction between the NH_2_ groups of the dendrimer and the COOH group of the medication, and the temperature of the dendrimer solution was shown to have an inverse relationship with the solubility of aceclofenac [102].

Likewise, Gautam and Verma investigated the effect of a full-generation PAMAM (G4) dendrimer on the solubility of candesartan cilexetil (lipophilic calcium channel blocker agent). Purified water was used throughout the testing, which was conducted at room temperature, and the drug’s concentration was determined to be 2.63 g/mL. The maximum solubility of candesartan cilexetil increased approx. 373-fold at a 10 mg/mL PAMAM concentration, and it was shown that the enhancement in solubility relies on the concentration of the dendrimers [103]. Simvastatin was investigated with dendrimers by Kulhari et al. with the goal of assessing the effectiveness of three different G4 PAMAM dendrimers. It was found that PEGylated dendrimers had the highest solubilization (33-fold), followed by NH_2_- (23-overlap) as well as OH-ended (17.5-overlay) dendrimers. The solvency improved from 33.4 to 1093.25 mole/L with the introduction of 109.04 M (0.4%, *w*/*v*) PEGylated dendrimer complexes (i.e., 33-overlap) [104].

### 3.3. Nanosuspensions

Nanosuspensions are submicron colloidal dispersions of drug particles in an aqueous phase, colloidally stabilized with the aid of surfactants [105]. As an adjunct to lipidic systems, nanosuspensions are employed in the formulation of drugs that are insoluble in both water and organic solvents. Compounds with a high melting point, a high log *p* value, and high dosage strength are the best candidates to be formulated as nanosuspensions [106]. By delivering the nanosuspension orally or intravenously (IV), the rate of saturation of the active component increases and the optimal plasma level is more rapidly achieved. The size distribution of solid particles in nanosuspension ranges from 200 nm to 600 nm [107]. Aghrbi et al. developed cilostazol-incorporated nanosuspension in an attempt to enhance the in vitro solubility and dissolution rate via a wet milling method, and the results showed that at pH = 1.2, the particle size reduction significantly increased the maximum thermodynamic solubility of the drug (cilostazol) and had a two-fold improvement over unmilled and pure surfactant dispersion [108]. Albendazole exhibits a solubility of 4.1 mg/L at 25 °C in water and <5% bioavailability through the oral route. Thus, to improve the solubility of albendazole, Rao et al. formulated nanosuspension-encapsulated multiparticulates; they found in their studies that there was a 16-fold increase in aqueous solubility [109].

### 3.4. Micelles

The combination of a hydrophilic spherical shell composed of polar heads or a hydrophobic core composed of a polar tail produces an optimal environment for the solubilization of poorly water-soluble drugs [86,110]. Polymeric micelles, which are typically composed of amphiphilic block copolymers, have received a lot of interest in the last few decades in terms of delivering hydrophobic payloads. Polymeric micelles with central hydrophobic sections composed of hydrophobic moieties, such as poly (propylene oxide) (PPO), PCL, poly (ethylene imine) (PEI), PLA, poly (jasmine lactone) (PJL), and phosphatidylethanolamine (DSPE), and an outer hydrophilic shell (typically poly (ethylene oxide) (PEO) may self-assemble into micelles in an aqueous media with low critical micellar concentration (CMC) [111,112]. Due to the particular features of polymeric micelles, such as nanoscale size, distinctive structure, stability, and compatibility, they are suitable for many types of applications [113].

Bansal et al. has evaluated the solubilizing capability of poly-based (jasmine lactone) (PJL) polymeric micelles against Soluplus^®^ and with poly (lactide) copolymer micelles. They found that after the introduction of -COOH groups to the polymeric chain of PJL, the aqueous solubility of clotrimazole was enhanced approx. 334-fold compared to Soluplus^®^. Hence, it was proposed that the solubilization capability of polymeric micelles can be enhanced drastically via the introduction of a free functional group on the polymer chain, which can interact with drugs electrostatically [114,115]. Zhou et al. synthesized griseofulvin-loaded core crosslinked micelles to increase solubility and stability. As early dissociation might be harmful to the micelles, linear dendritic polymers were crosslinked in this study to avoid both it and drug leakage. The results of the study suggest a 10-fold improvement in the solubilization and sustained-release behavior of griseofulvin in developed polymeric micelles [116].

### 3.5. Solid Lipid Nanoparticles and Nanostructured Lipid Carriers

Solid lipid nanoparticles (SLNs) and nanostructured lipid carriers (NLCs) are two lipid-based nano-systems that have sparked a great deal of interest for a method of orally delivering hydrophobic drugs with low bioavailability [117]. These lipid nanocarriers offer several advantages including biocompatibility, simplicity of scale-up, enhanced lymphatic transport, and, hence, lower first-pass metabolism. SLNs are the 1st generation of lipidic nanoparticles, measuring around 50 to 1000 nm in diameter, and are composed of an aquatic lipid matrix dispersion (composed of approximately 0.1–30 (% *w*/*w*) solid fat dispersed in an aqueous phase) stabilized using surfactants, which is solid at both room and body temperatures [117]. Nevertheless, these systems have several disadvantages, which include inefficient drug loading and the potential for drug leakage [118]. NLCs, similar to SLNs, are innovative SLNs constructed out of solid lipids and liquid lipids. A larger payload, reduced drug leaching during storage, and improved performance in producing the final dosage forms, such as creams, tablets, capsules, and injectables, can be achieved. Moreover, suspensions of higher solid content (e.g., 30–50% solid) and the sustained release of medication are the advantages of NLCs. These liquid oils inside the solid–lipid matrix provide a matrix with a much lower lipid content, which allows for more cargo molecules to be accommodated. For the oral administration of hydrophobic drugs, several investigations have looked into SLN or NLC formulations [119]. Hu et al. developed solid lipid nanoparticles (SLNs) to improve the oral bioavailability of all-trans retinoic acid (ATRA), a poorly soluble drug used as the model drug, and the findings demonstrated that the absorption of ATRA is improved significantly by incorporating it into SLN formulations [120]. Khan et al. evaluated the potential of NLCs for the improvement of the solubility and bioavailability of tacrolimus (TL). He and his colleagues created a tacrolimus-loaded nanostructured lipid carrier for this purpose, and they discovered that it increased the relative bioavailability of TL-NLC by 7.2 times compared to TL suspension [121].

### 3.6. Supercritical Antisolvent (SAS)

SAS is far more effective than liquid solvent precipitation and may be utilized as a distinctive, environmentally friendly technique for producing nanomaterials. Supercritical CO_2_ has been widely used to create a wide range of materials, including polymers, biopolymers, superconductors, explosives, colorants, active pharmaceutical ingredients (APIs), and catalysts. If the processed compounds do not dissolve in the supercritical medium, an antisolvent is used to cause the controlled precipitation of solids to be dissolved in the conventional solvent. These chemicals dissolve in an organic liquid that is miscible with the supercritical antisolvent in the proper processing conditions. In order to produce extremely porous nanoparticles, SAS combines the advantages of the sol-gel method with the use of a supercritical CO_2_ antisolvent [122]. Conventional micronization techniques such as milling, grinding, and spray drying, which rely on mechanical and thermal stress to disaggregate the active compound, have the drawbacks of the overuse of solvent, the thermal and chemical degradation of pharmaceuticals, polymers, and biologically active proteins, a high concentration of residual solvent, and, most importantly, difficulty in controlling the particle size and distribution during processing. These disadvantages can be overcome via processes based on the use of supercritical fluids, the most common of which is supercritical carbon dioxide (scCO_2_). The SAS process is predicated on a few key prerequisites. Because scCO_2_ serves as an antisolvent in this technique, it must be completely miscible with the liquid solvent used. In the literature, the SAS process is most frequently used to create microparticles that increase APIs’ aqueous solubility. Rapid contact between the two media (i.e., antisolvent and polymer/drug solution) speeds up the process of nucleation and growth, resulting in the formation of very fine particles that gives SAS an advantage over all abovementioned conventional methods [123].

**Figure 6:**
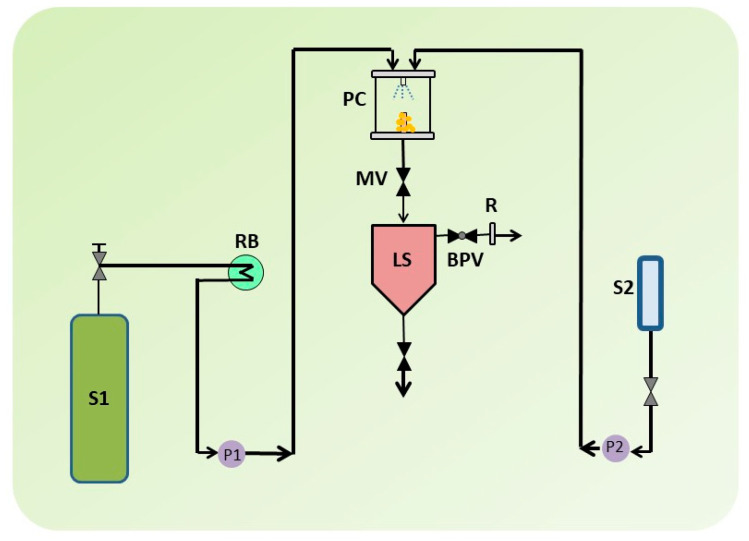
A schematic illustration of supercritical antisolvent (SAS) apparatus. S1: CO_2_ supply; S2: Liquid solution supply; P1 & P2; Pumps; RB: Refrigerating bath; LS: Liquid Separator; PC: Precipitation Chamber; MV: Micrometric Valve; BPV: Block-pressure Valve; R: Rotameter.

Traditional procedures can benefit from novel, more effective micronization techniques such as SCF-assisted particle formation, which can produce solvent-free products in comparatively normal circumstances. Supercritical antisolvent (SAS) methods have the capacity to improve the solubility and bioavailability of a number of medications that are poorly water soluble [123]. Although SCFs are incredibly dense and generally organic solvents are miscible with it, the essential mechanism of the SAS technique is dependent on critical parameters such as temperature, pressure, nature of the solvent, flow rate, and nozzle geometry [123,124]. A SAS experiment begins by pumping CO_2_ into the precipitator, which is then heated to the desired temperature. After the operating conditions have been stabilized, the pure solvent is delivered to the precipitator via a nozzle. The liquid solution containing the solute/solutes dissolved in the chosen solvent is then injected. The solute(s) precipitates on a filter as a result of supersaturation. The solvent/antisolvent mixture is recovered and separated downstream of the precipitator, where a vessel to collect the liquid solvent is located. After injecting the solution, the scCO_2_ continues to flow to eliminate the solvent residues. The precipitator is depressurized to atmospheric pressure at the end of this washing step, and the precipitated powder can be collected [125]. The critical conditions when SCFs are extremely dense and usually miscible with organic solvents determine the basic mechanism of the SAS process. Figure 6 displays a schematic of the SAS process.

Telmisartan is a BCS class II drug with severely limited solubility in water, but it is easily soluble in strongly alkalized solutions. Telmisartan dissolves in only a few organic solvents. The most significant barrier to reaching the desired bioavailability is the issue of solubility. The SAS technique was utilized to micronize, amorphize, or solid disperse BCS class II drugs in several ways due to its unique properties. The SAS technique has been used for producing solid dispersions of hydroxy propyl methyl cellulose/polyvinylpyrrolidone (HPMC/PVP) at 1:0.5, 1:1, and 1:2 weight ratios of drug to polymer, while pure telmisartan had also been processed. In conformity with the researchers, this SAS technique might be a potential approach for accelerating the dissolving or improving the solubility of telmisartan after adjusting the solid dispersion formulation [126]. Likewise, in order to study fluconazole monohydrate using the SAS method, Park et al. varied the temperature (40, 60, and 80 °C), the pressure (8, 12, and 16 MPa), and the type of solvent (acetone, ethanol, and dichloromethane (DCM)). At high pressure, neither particle precipitation nor nucleation took place (16 MPa). Nevertheless, both were observed when the pressure was being reduced (12 MPa). This is caused by the increased solubility of fluconazole in SC-CO_2_ at high pressures. Additionally, the product yield increased gradually as the temperature was raised from 40 to 80 °C while maintaining the same pressure. At high temperatures, the organic solvent’s solubility in SC-CO_2_ increased, causing the solvent to be extracted more quickly, aiding in the drug’s precipitation [127].

### 3.7. Nanoemulsions

Nanoemulsions are heterogeneous, thermodynamically stable systems consisting of an oil phase and an aqueous phase with one dispersed in the other with the assistance of the surfactant, where the interfacial film formation of the surfactant provides colloidal stability to the system [128]. Having a droplet size lower than other that of colloidal systems (1–100 nm) confers a greater surface area (e.g., standard emulsions), which assists in improving solubility [129]. Reddy et al. has formulated nanoemulsions of febuxostat, a BCS class II drug used for solubility enhancement. The outcome of their in vitro dissolution study demonstrated that within 6 h, 42.37% of the drug was released from the formulated nanoemulsion; this indicated that the improved solubility of the drug is due to the developed formulation [130]. Ostwald ripening, a foremost destabilization process of nanoemulsions, is a process in which larger droplets grow at the smaller droplets’ expense. This process can be avoided or slowed down by using hydrophobic components in the oil phase followed by a decreased rate of coalescence. Wik et al. prepared a nanoemulsion having an oily phase of a renewable poly (δ-decalactone) (PDL) and Pluronic F-68 as a surfactant via the nanoprecipitation method for the evaluation of drug delivery potential using various hydrophobic drugs. They found that, compared to well-founded Pluronic micelles, the developed nanoemulsion (with droplet size < 200 nm) enhanced the aqueous solubility of the drugs by improving it from 3- to 10-fold [131,132].

### 3.8. Nanogels

Nanogels are three-dimensional hydrogel substances with a high capacity to hold water and are generated by crosslinked swellable polymer networks in the nanoscale size range without physically dispersing into the aqueous media [133]. Nanogels are created by physically or chemically crosslinking nanoscale-sized networks, which include networks made of neutral and cationic polymers such as poly (ethylene glycol) (PEG) and polyethylenimine (PEI) [134]. Particle sizes in nanogels range from 100 to 200 nm [135], and altering the solvent quality assists in maintaining the three-dimensional network of the nanogel [136]. They have drawn significant attention as versatile polymer-based nanodrug delivery systems, as they are capable of encapsulating both hydrophilic and hydrophobic molecules. Having large surface areas, good drug loading capacities, and effectiveness for the solubility augmentation of poorly soluble drugs makes nanogels a promising, effective, and safe nanotechnological approach for delivering drugs [137]. Yao et al. developed myricetin (flavonoid) loaded novel nanogel based on chitosan. This strategy promoted a 2.20-fold increase in oral bioavailability compared to plain myricetin in rats [138]. Khan et al. has designed a nanogel system in an attempt to enhance the solubility of olanzapine (OLZ), an antipsychotic drug, via the crosslinking of Poloxamer-407 and 2-acrylamido-2-methylpropane sulfonic acid (AMPS) with the assistance of methylene bisacrylamide (MBA). The results confirmed that in comparison to the free drug, the solubility of olanzapine in nanogel formulation was improved by up to 38 times [139].

### 3.9. Metal Organic Frameworks (MOFs)

Metal-organic frameworks (MOFs), which integrate organic ligands with metal ions or metal complexes via coordinative bonding to form a two-dimensional or three-dimensional network, are very porous and crystalline materials that would provide molecular structural flexibility [140]. Due to their customizable physiochemical characteristics (i.e., surface area, modulable porosity, functional moieties, tunable pore size, and pore volume and flexibility to encapsulate significant active ingredient loadings), MOFs have attracted a lot of consideration as drug delivery carriers in the past 10 years [141]. MOFs are an excellent representation of the ability to merge organic and inorganic chemistry, two areas that are sometimes seen as incompatible [142].

Quercetin (Que) exhibits multifunctional pharmacological properties, which include anti-cancer, anti-hypertensive, and antioxidant activities, but is associated with very poor water solubility. In an attempt to overcome this limitation, Wang et al. loaded Que into γ-cyclodextrin metal-organic frameworks (γ-CD-MOFs) in which they observed the 100-fold enhancement of solubility relative to pure Que [143]. One of the promising solutions by Chen et al. for isosteviol’s (STV) insolubility is the use of the highly porous supramolecular carrier cyclodextrin’s metal-organic framework (CD-MOF). STV’s solubility in water was less than 20 ng/mL at a pH of 1.0 and a pH of 4.5, but it was more soluble at a pH of 6.8 and 129.58 ng/mL; therefore, it exhibits pH dependency. The bioavailability of STV@CD-MOF (1:1) was 8.67 times greater than that of STV, 1.32 times greater than that of STV@CD, and 1.27 times greater than that of STV@CD-MOF (0.5:1) in rats [144]. Similarly, to enhance the solubility of Azilsartan (AZL), an angiotensin II receptor antagonist, He et al. designed a γ-CD metal-organic framework (γ-CD-MOF). AZL was effectively confined in biocompatible versatile γ-CD-MOF high molecular cages, resulting in clusters in the nanometer range, improving solubility. Using this method, when compared to the pure drug, the relative solubility of AZL/CD-MOF increased by 340 times; the bioavailability of AZL increased by 9.7 times after loading into the CD-MOF observed in Sprague-Dawley rats [145].

### 3.10. Carbon Nanotubes

Carbon nanotubes (CNTs) are a promising carrier in nanotechnology with peculiar electrical, mechanical, chemical, and optical properties. They are cylinder-shaped, allotropic forms of carbon. On the surfaces of CNTs, functional groups are created via functionalization. These functional groups aid in the enhancement of the contact between the CNTs and the matrix or solvent and produce a homogenous dispersion or cause the solubilization of the CNTs. To avoid aggregation and improve their dispersibility, the surface modification of the CNTs is necessary for better interactions with matrix materials and polymer matrices [146,147]. Due to the high water dispersibility of functionalized CNTs, they can serve as the drug’s nucleating sites for hydrophobic compounds and enhance hydrogen bonding with aqueous media, which facilitates fast dissolution [148]. Therefore, the underlying mechanism by which CNTs improve the solubility of drugs that are weakly water-soluble is known as “functionalized partitioning” [149]. Chen et al. introduced CNTs into hydrophobic drugs (griseofulvin and sulfamethoxazole) during synthesis. The results demonstrated that the carrier enhances the dissolution rate of both pharmaceuticals. For CNTs in griseofulvin (4%), it takes 18 min instead of 66 min. For CNTs in sulfamethoxazole (5.1%), it takes 10 min instead of 67 min to obtain 80% dissolution [150]. Further, Zhu et al. formulated dipyridamole CNTs, as it is a poorly soluble drug, and concluded an increase in drug loading; the form of dipyridamole changed from amorphous to crystalline. Moreover, as drug loading into carriers improved, the release rate of the drug dropped and improvement in dissolution rate was perceived. CNTs have been also shown to be promising carriers for loading dipyridamole [151].

### 3.11. Mesoporous Silica

Mesoporous silica has been extensively recognized for possessing the ability to improve solubility by adsorbing and thereby stabilizing APIs in their amorphous state within their porous network [152,153]. Consequently, it has been suggested that mesoporous silica materials (MSMs) be employed as matrices to increase the apparent solubility and dissolution rate of poorly water-soluble drug molecules. Because amorphous silica is a “generally regarded as safe” (GRAS) material, biodegradable by hydrolysis, and easily surface-modifiable to enhance drug loading and subsequent release in the human body, mesoporous silica materials are excellent candidates for drug delivery [154]. The primary benefits of mesoporous silica as drug delivery systems for poorly water-soluble drugs are their pore size, pore morphology, and versatility in altering the surface chemistry; the latter can result in optimized interactions between a drug candidate and the mesoporous silica carrier by modifying the pore surfaces [155]. The fundamental property associated with MSMs in this regard is nevertheless their characteristic pore size, which per the IUPAC definition lies in the mesoporous range (2–50 nm). Namely, Rengajaran et al. were able to derive that molecules residing in pores less than 10 times their size remains in amorphous forms due to not having the space to form crystals [156]. This phenomenon has been utilized in the formulation of orodispersable films (prednisolone) [157], fast dissolving tablets (tamoxifen) [158], and lyophilized tablets (silymarin) [159,160]. In one in vivo study, spherical mesoporous silica nanoparticles (MSNs) were developed by Zhang et al. as an oral drug delivery system to enhance the oral bioavailability of the drug telmisartan (TEL). Model drug permeability tests in the human colon cancer (Caco-2) cell lines showed that MSNs may significantly increase TEL permeability and decrease the rate of drug efflux. The oral bioavailability of TEL-laden ordered MSMs, MSNs, and the commercial drug Micardis were investigated in beagle dogs. They found in their studies that the TEL-loaded MSNs formulation had a relative bioavailability of 154.4 ± 28.4% and the TEL-loaded MSMs formulation had a relative bioavailability of 129.1 ± 15.6% [161]. A similar study was later repeated in a clinical setting by Bukara et al. in which they showed that both the absorption rate and the extent was significantly enhanced for fenofibrate loaded into MSMs vs. a marketed micronized formulation [162]. This study served as the first form of evidence for this relatively novel formulation approach.

**Table 5:** Summary of various delivery systems and polymers used for the solubilization of different drug molecules.

Delivery System/Method Employed	Polymer Used	Drugs/API	Structure	Details	References
PAMAM Dendrimer	Amine and ester-terminated PAMAM Dendrimers	Nifedipine	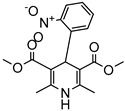	Dendrimers composed of poly (amidoamine), or PAMAM, can improve the solubility of insoluble drugs in water at pH 7.	[163]
	Dendrimers made of polyamidoamine(PAMAM) G3.5 and PAMAM G4.5.	Oxaliplatin	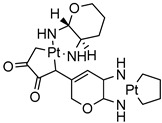	The solubility of oxaliplatin increases roughly linearly with dendrimer concentration.	[164]
	Dendrimers made from PAMAM	Temozolomid	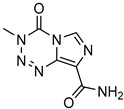	TMZ solubility was shown to be enhanced in some solvent systems, with dendrimer, ethanol, and tween-20 showed construction and related in solubility.	[165]
	PAMAM dendrimers with pyrrolidone modification	Indomethacin	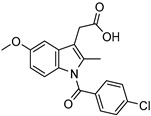	The drug’s solubility and intracellular delivery are being improved	[166]
	PAMAM dendrimers	Nicotinic acid	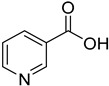	PAMAM dendrimers of different generations (G1–G4) have the ability to dramatically improve nicotinic acid solubility.	[167]
	Polyether dendrimer	Artemether	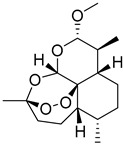	Due to their excellent water solubility, non-immunogenicity, and increased biocompatibility, they are used as drug carriers.	[168]
	PAMAM dendrimers	Ibuprofen	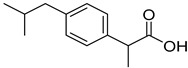	PAMAM dendrimers improve ibuprofen solubility much more than SDS micelles.	[169]
	PAMAM and Lauryl PAMAM dendrimer	Propranolol	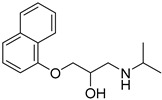	Propranolol’s solubility has been improved	[170]
Silica	Cur-fls & Cur-sls	Curcumin	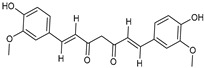	Improved solubility with enhanced oral bioavailability up to 7-fold high than convectional suspensions.	[171]
Thin film hydration sonication	Glycol, Eudragit S100	Sorafenib	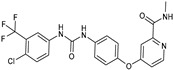	It improved systemic exposure of about four-fold.	[172]
Thin-film hydration sonication	Lecithin	Cefotaxime	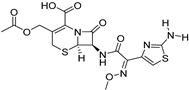	About five-fold increase of in oral bioavailability and improved solubility	[173]
Thin-film hydration sonication	Soy lecithin	Capsaicin	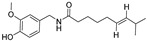	Oral bioavailability and improved solubility increase about three-fold.	[174]
Film deposition on the carrier	HSPC	Lopinavir	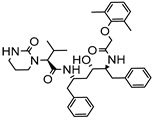	Improved solubility with enhanced oral bioavailability up to 2-fold.	[175]
Thin-film hydration sonicate	DSPC	Asinine maleate	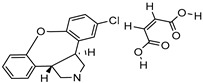	About one-fold increase in oral bioavailability and improved solubility	[176]
Thin-film hydration sonication-freeze thawing	SPC	Spironolactone	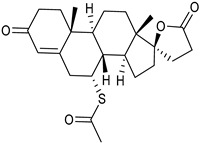	Enhanced oral bioavailability with improved solubility up to 2-fold.	[177]
High-pressure homogenization	Poly Na styrene sulfonate	Paclitaxel	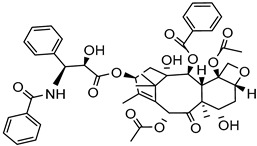	About 14 -fold increase in oral bioavailability and improved solubility and drug dissolution: 20% (120 min).	[178]
Antisolvent precipitation	Pluronic^®^ F68	Puerarin	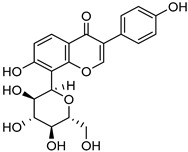	Enhanced oral bioavailability with improved solubility up to 4-fold	[179]
Spray drying	SDS	Alisertib isoproxil	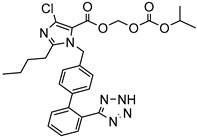	Drug dissolution: ~14% (1/2 min) enhanced oral bioavailability with improved solubility up to 4-fold.	[180]
Antisolvent precipitation	Ethyl cellulose	Domperidone	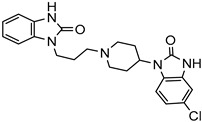	Fifty percent (30 min) and 65 percent (60 min) drug dissolution and enhanced oral bioavailability with improved solubility up to 2-fold.	[181]
Precipitation-sonication	PVA	Cinnarizine	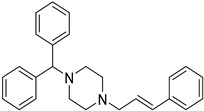	One hundred percent drug in dissolution (240 s) enhanced oral bioavailability with improved solubility up to 2-fold.	[182]
Magnetic stirring-milling	PVP-K30	Glyburide	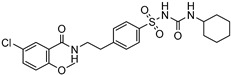	100 percent drug in dissolution (30 min) increased oral bioavailability with improved solubility up to four-fold.	[183]
Hot homogenization sonication	Stearic acid	Rosuvastatin	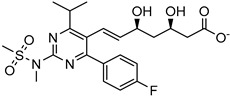	Drug release: ~45 percent (120 min) and ~80 percent (10 h) Improved oral bioavailability with improved solubility up to 8-fold.	[184]
Micro emulsification	Compritol	Rifampicin	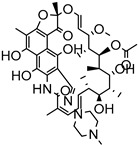	Enhanced oral bioavailability with increased solubility up to 8-fold.	[185]
Emulsification sonication	Precirol^®^ ATO-5, palmitic acid, Gelucire^®^ 50/13, N	Resveratrol	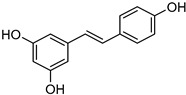	Enhanced oral bioavailability and having a higher level of solubility up to 7-fold.	[186]

## 4. Conclusions

This review offers a critical assessment of previously reported literature and some newly emerging technologies, which include formulation design, solid particle techniques, prodrug strategies, micronization, solid dispersions, particle size reduction technologies, nanosizing, cyclodextrins, solid lipid nanoparticles, drug conjugates, colloidal drug delivery systems, nanoemulsion, micelles, and so forth. Most new drug molecules entering the development pipeline are poorly water-soluble. Solubility is an important parameter for successful formulation development, as it directly controls the bioavailability, defines what kind of formulation should rationally be attempted, and affects the therapeutic efficacy of the drug. The nanotechnological approach is another strategy that has sparked interest in drug solubility enhancement, being one of the easiest tasks associated with nanomedicines. They have been used as platforms for enhancing drug solubility. Despite the fact that nanotechnology has made significant strides, some difficulties have been witnessed in the expansion of novel drug delivery systems. These difficulties include the transition of these nanocarriers from the laboratory to the pharmaceutical market, relying on factors such as fabrication costs and the reproducibility of formulation properties on the production scale, and benefits to the human population because of the divergent pharmacokinetics profile. Nevertheless, despite these difficulties, innovative drug delivery technologies continue to advance and provide benefits that cannot be overlooked. As a result, nanotechnology provides formulation scientists with a chance to expand their research and development in order to address the problems associated with poorly soluble drugs, consequently increasing the therapeutic efficacy. Solving the above-mentioned problems is based on molecular properties, but researchers anticipate that solid dispersions and lipid delivery will be the most sought-after techniques to solve these issues in a reasonable number of drug compounds.

## 5. Challenges and Future Perspectives

The growing array of insoluble APIs that have been discovered over the past few decades has intensified the burden on scientists to develop and adopt innovative techniques to improve bioavailability. With the goal of delivering effective pharmaceuticals products, scientists need to investigate more varied formulation approaches to overcome the formulation challenges. Despite the existence of an assortment of conventional approaches that can boost bioavailability, additional research is needed to create practical and efficient formulation approaches. Even though solid dispersion techniques and lipid-based nanotechnological approaches have been the focus of recent research, there are still relatively few commercially available products employing these techniques due to issues with production scale-up, physicochemical instability, short expiry periods, and reproducibility issues. To assist with poorly soluble molecules, a number of trends in solubility enhancement have been developed, including novel methods and excipients. However, applying a completely new methodology is an expensive affair for the pharmaceutical industry, and it often requires new infrastructure and personnel. To simplify the process and to identify the best approach, attention has been directed to molecular modelling. Although this is not yet possible, scientists are moving closer to being able to use molecular dynamic simulations to determine which excipients or technologies will function most effectively.

For instance, Fagerholm et al. suggested a novel approach where oral bioavailability in humans is predicted from a chemical structure by directly utilizing an integrated technique comprising 9 machine learning models, 3 sets of structural alerts, and 2 physiologically based pharmacokinetic models. On a benchmark dataset of 184 chemicals, they evaluated the model and achieved a predicted accuracy (Q2) of 0.50; which is considered successful by the pharmaceutical sector. They arrived to the conclusion that this approach has enough predictive accuracy to be feasible in applications predicting human exposure and dose, compound optimization, and decision-making, with potential to rationalize drug discovery and development and reduce failures and overexposures in early clinical trials with candidate drugs [187]. Since administering medications orally is the most practical method, it is essential to accurately determine oral bioavailability during drug discovery and development. Quantitative structure-property relationship (QSPR), rule-of-thumb (RoT), and physiologically based-pharmacokinetic (PBPK) approaches are promising alternatives for early oral bioavailability prediction [188]. Combining the power of artificial intelligence and novel solubility enhancement technologies could be a game changer in solving solubility issues, which is also capable of reducing R&D cost.

## Figures and Tables

**Table 1:** BCS Classification.

BCS Class	Solubility	Permeability	Drug Molecule Examples
I	High	High	Mefoquine hydrochloride, Nelfnavir mesylate, Quinine sulfate, Clomiphene citrate
II	Low	High	Ibuprofen, Nifedipine, Carbamazepine, Diazepam, Efavirenz
III	High	Low	Amiloride hydrochloride, Amoxicillin, Ethosuximide, Fluconazole, Isoniazid, Salbutamol
IV	Low	Low	Acetazolamide, Dapsone, Doxycycline, Nalidixic acid, Theophylline

**Table 2:** Factors affecting solubility.

S. No	Factors Affecting Solubility	Details	References
1.	Particle size	As particle size is reduced, the surface area will increase, and the larger surface area will provide a greater interaction of the solute molecules with the solvent.	[34]
2.	Temperature	Solubility will be increased when the temperature rises and the solution process absorbs energy; if the solution process generates energy, then solubility will decrease with an increase in temperature.	[35]
3.	Pressure	Pressure will only affect the solubility of gaseous solutes and have no effect on solid and liquid solutes. A decrease in pressure causes a decrease in solubility, and an increase in pressure causes an increase in the solubility of gaseous solutes.	[35]
4.	Nature of solute and solvent	Properties of solute, as well as the solvent, have drastic effects on solubility.	[36]
5.	Polarity	Substances with the same type of polarity will be soluble in one another, “similia similibus solvuntur”. Polar solute molecules or ions will dissolve in polar solvents, while non-polar solute molecules will dissolve in non-polar solvents.	[37]
6.	Polymorphism	Polymorphs differ in melting points. Different polymorphs have different solubilities as solubility and melting point are linked.	[38]
7.	Stirring	Stirring ensures that new solvent components come into contact with the solid and liquid solutes, resulting in increasing solubility.	[39]

**Table 3:** A list of several commercial solid dispersion products of poorly soluble drugs.

S. No	Trade Name	Therapeutic Agent	Manufacturer	Polymer Used in Formulation	Indication
1.	Certican	Everolimus	Novartis	HPMC	Anti-cancer
2.	Cesamet	Nabilone	Valeant Pharmaceuticals	PVP	Chemotherapy-induced nausea
3.	Gris-PEG	Griseofulvin	Pedinol Pharmacal Inc.	PEG6000	Antifungal
4.	Intelence	Etravirin	Tibotec	HPMC	Antiviral (HIV infection)
5.	Isoptin SR-E	Verapamil	Abbott	HPMC/HPC	Anti-Hypertensive
6.	Nivadil	Nivalidipine	Fujisawa Pharmaceutical Co., Ltd.	HPMC	Anti-Hypertensive
7.	Prograf	Tacrolimus	Fujisawa Pharmaceutical Co., Ltd.	HPMC	Immunosuppressant
8.	Rezulin	Troglitazone	Pfizer, Inc.	PVP	Antihyperglycemic
9.	Sporanox	Itraconazole	Jansen Pharmaceuticals, Inc.	HPMC	Antifungal

**Table 4:** An assessment of advantages and disadvantages of various conventional and nanotechnological solubility enhancement techniques.

S. No	Techniques	Advantages	Disadvantages	References
1.	Particle Size Reduction	Increases surface area volume ratio	Due to the high surface charge on discrete small particles, there is a strong tendency for particle agglomeration. Thermal stress may occur, which harms thermosensitive or unstable active compound.	[74]
2.	Cyclodextrin Inclusion Complex	Cyclodextrin has high aqueous solubility and commensurately low viscosity. High API concentrations are achievable. Additionally, facilitates chemical stability.	Cyclodextrins demonstrates renal toxicity in most species, limiting their use in pre-clinical toxicology assessments.	[75,76]
3.	Solid Dispersion	Dissolution rate and bioavailability are enhanced by keeping drug in more soluble amorphous state.	Not commonly used as a commercial product because of the conversion of the amorphous drug into the less soluble crystalline form on long-term storage and, consequently, increased drug mobility can lead to phase separation and instability. Large-scale production is limited due to expensive preparation methods.	[75]
4.	Prodrug approach	Higher solubility in lipid membranes and improved oral or local absorption. Reduced toxicity and local irritation. Increases chemical or metabolic stability.	Not feasible for all drug formulation.	[74]
5.	Supercritical fluid technology	Free from organic solvents and heavy metals. Green extraction techniques.	Expensive and complex equipment, operating at elevated pressure. High power consumption.	[68]
6.	Polymeric Micelles	Ease of fabrication and chemical modification. Suitable for numerous hydrophobic drug candidates. Control and targeted drug release is possible.	The disintegration of micelles due to their dilution after oral administration, in vivo instability below the critical micellar concentration. Low drug loading.	[77]
7.	Polymeric Nanoparticles	Enhanced drug stability, sustained drug delivery, shielding of the drug cargo from enzymatic activity, prolonged retention in the GI tract, and improved mucoadhesiveness.	Challenges in biocompatibility and safety of polymeric carriers. Toxicity is a result of the high tissue accumulation of non-biodegradable NPs. Difficulties in optimizing the process parameters and scaling up the production into a pharmaceutical product.	[78]
8.	Liposomes	Non-immunogenic, biocompatible, and biodegradable. Ability to carry both hydrophilic as well as hydrophobic drugs.	Poor stability and short shelf life.	[79]
9.	Solid lipid nanoparticles (SLNs)	Biocompatible. Easy scale-up. Protects drug against harsh environmental conditions.	Because of crystalline structure, low drug-loading efficacy and chance of drug expulsion during storage.	[80]
10.	Dendrimers	Drug encapsulation and conjugation is possible. Tunable chemical and physical properties.	May cause cellular toxicity. Elimination and metabolism depending on the generation of the dendrimers. High synthetic cost.	[77]
11.	Quantum dots	Multiple molecular targets simultaneously.	Toxicity effect of metal core.	[81]
